# Interactive SARS-CoV-2 dashboard for real-time geospatial visualisation of sewage and clinical surveillance data from Dhaka, Bangladesh: a tool for public health situational awareness

**DOI:** 10.1136/bmjgh-2023-012921

**Published:** 2023-08-24

**Authors:** Erin G Wettstone, Md Ohedul Islam, Lauren Hughlett, Claire Reagen, Tahmina Shirin, Mahbubur Rahman, Kawsar Hosan, Md Raihanul Hoque, Stephanie A Brennhofer, Elizabeth T Rogawski McQuade, Yoann Mira, Lukas von Tobel, Rashidul Haque, Mami Taniuchi, Isobel M Blake

**Affiliations:** 1 Division of Infectious Diseases & International Health, University of Virginia, Charlottesville, Virginia, USA; 2 Department of Biomedical Engineering, University of Virginia, Charlottesville, Virginia, USA; 3 Infectious Diseases Division, International Centre for Diarrhoeal Disease Research Bangladesh, Dhaka, Bangladesh; 4 Institute of Epidemiology, Disease Control and Research, Dhaka, Bangladesh; 5 Department of Economics, Jahangirnagar University, Dhaka, Bangladesh; 6 a2i, Dhaka, Bangladesh; 7 Department of Epidemiology, Emory University, Atlanta, Georgia, USA; 8 Novel-T, Geneva, Switzerland; 9 Department of Civil and Environmental Engineering, University of Virginia, Charlottesville, Virginia, USA; 10 MRC Centre for Global Infectious Disease Analysis, School of Public Health, Imperial College London, London, UK

**Keywords:** COVID-19, SARS, Public Health, Epidemiology

## Abstract

Throughout the COVID-19 pandemic, many dashboards were created to visualise clinical case incidence. Other dashboards have displayed SARS-CoV-2 sewage data, largely from countries with formal sewage networks. However, very few dashboards from low-income and lower-middle-income countries integrated both clinical and sewage data sets. We created a dashboard to track in real-time both COVID-19 clinical cases and the level of SARS-CoV-2 virus in sewage in Dhaka, Bangladesh. The development of this dashboard was a collaborative iterative process with Bangladesh public health stakeholders to include specific features to address their needs. The final dashboard product provides spatiotemporal visualisations of COVID-19 cases and SARS-CoV-2 viral load at 51 sewage collection sites in 21 wards in Dhaka since 24 March 2020. Our dashboard was updated weekly for the Bangladesh COVID-19 national task force to provide supplemental data for public health stakeholders making public policy decisions on mitigation efforts. Here, we highlight the importance of working closely with public health stakeholders to create a COVID-19 dashboard for public health impact. In the future, the dashboard can be expanded to track trends of other infectious diseases as sewage surveillance is increased for other pathogens.

**Summary box**

- Systematic sewage surveillance for SARS-CoV-2 has been shown to be an effective early warning indicator of impending increases in COVID-19 cases.
- Although sewage surveillance systems have been established in multiple countries, interpretation of SARS-CoV-2 sewage data has been difficult in isolation of clinical COVID-19 incidence data.
- Here, we present an example of a dashboard that presents real-time visualisation of both COVID-19 clinical case data and SARS-CoV-2 sewage surveillance data for a megacity in a lower-middle-income country, which has been developed through integrating feedback from public health officials.
- The dashboard is a powerful tool that makes real-time data interpretable to government stakeholders and the public. It provides a comprehensive picture of the community burden of COVID-19 in a city where clinical testing is limited.
- If sewage surveillance expands to monitor more infectious diseases, similar dashboards integrating geospatial environmental and clinical surveillance data will be important to visualise real-time transmission of infectious diseases for public health stakeholders.

## Introduction

COVID-19 has killed millions and infected hundreds of millions since the global pandemic began in the winter of 2019.^1^ Clinical testing for SARS-CoV-2, the aetiological infection, is the gold standard for tracking COVID-19 incidence, but there are many barriers to clinical testing, especially for low-income and lower-middle-income countries.^2^ Low-income and lower-middle-income countries often lack the resources to roll out widespread COVID-19 testing or the ability to organise regular testing due to high-density population or remote living conditions.^3 4^

Systematic sewage surveillance for SARS-CoV-2 has been employed by many governments as a complementary source of COVID-19 tracking and has been shown to capture the temporal dynamics of SARS-CoV-2, providing an early warning of increasing transmission.^5–7^ We have shown that sewage surveillance in Dhaka, Bangladesh, can act as an early warning indicator for COVID-19 cases up to 2 weeks in advance of large rises in COVID-19 cases and it provided evidence of persistent circulation of SARS-CoV-2 in areas with low access to clinical testing.^8^ Sewage surveillance can also capture both asymptomatic and symptomatic cases, while clinical surveillance largely captures symptomatic cases.^6^ This is important for diseases such as COVID-19, where up to 40% of the infections are asymptomatic and many individuals go untested.^9^ Additionally, sewage surveillance is unbiased to health-seeking behaviour^6^, is less expensive and is more efficient because one sample can capture the burden across hundreds and thousands of people. Hundreds of sewage surveillance sites have opened around the world to track SARS-CoV-2, but there has been incomplete reporting of sewage surveillance data and sharing it with the public and other researchers.^10^

Dashboards have become an increasingly popular way to report COVID-19 incidence to government officials and to the public.^11–13^ A dashboard tool provides longitudinal and geospatial visual graphics to relate public health information to the end user. Dashboards such as the Johns Hopkins dashboard became a central tool for the public to check global COVID-19 statistics as the pandemic progressed.^14^ Researchers have also created dashboards that report sewage surveillance data.^10 15^ However, a recent review of sewage surveillance found its reporting to be incomplete: of the 200 universities, 1400 sites and 55 countries that report SARS-CoV-2 surveillance, only 59 dashboards are publicly available.^10^ Moreover, the number of low-income and lower-middle-income countries conducting sewage surveillance is disproportionately low (possibly due to the large upfront investment in mapping informal sewage systems prior to commencing surveillance and the challenges of obtaining adequate operational funding) and hence few dashboards have been developed in these settings.^10^ Additionally, many dashboards that report sewage surveillance data do not incorporate clinical case incidence.^15^ Combined reporting of both data sets allows public health officials to know where under-reporting of cases occurs and ensures that sewage collection sites are tracking the correct population.

Our sewage surveillance dashboard for SARS-CoV-2 in Dhaka, Bangladesh provides a comprehensive visualisation of both SARS-CoV-2 burden in sewage and COVID-19 clinical cases for a city in a lower-middle-income country. The goal of the dashboard was to enable effective dissemination and interpretation of sewage surveillance data with public health officials, which hence enabled the complementary surveillance system to be useful in improving situational awareness. The key objectives were to display both sewage and clinical data at a fine geographical resolution, given the large differences in socioeconomic status across the city, and to visualise the sewage catchments to inform which populations the sewage surveillance was representing. Here, we describe how the dashboard was developed and outline the key features that allowed the dashboard to be usable by public health officials.

## Development

### Stakeholder partnerships

The sewage surveillance dashboard for SARS-COV-2 in Dhaka, Bangladesh, began as a collaborative and shared development process across stakeholders from many backgrounds. The dashboard was designed for Dhaka and was created at the University of Virginia (UVA) in collaboration with Imperial College London and the International Centre for Diarrhoeal Disease Research, Bangladesh (icddr,b). Partnership and input from local government officials and public health professionals were imperative to the success of the dashboard. A collaboration was formed with individuals at the Institute of Epidemiology, Disease Control and Research (IEDCR), a Bangladesh government research institute under the national Ministry of Health, together with government officials on the national COVID-19 national task force. During the early stages (first 3 months) of development, the dashboard was sent to local stakeholders to review features such as navigation, digestibility, visual aesthetics and wording. After the dashboard became deployable, it was presented to stakeholders on a weekly basis in a report for the COVID-19 national task force, and feedback was given as the pandemic progressed and new ideas for improvement arose.

Through active dialogue with local public health officials and government stakeholders, we learnt about their need for visual features that would allow them to make clear inferences about the COVID-19 situation in Dhaka and the dashboard evolved through several iterations. The main requests of the stakeholders were to incorporate colour-coded severity levels for ease of interpretation and to overlay clinical case data and sewage surveillance data on a fine spatial scale for rapid interpretation of specific geographical areas in Dhaka, given the large differences in socioeconomic status of the population across the city. Several public health disclaimers were also added to the main mapping tab of the dashboard to prepare the dashboard for public use.

The dashboard has visualised data online in real-time since 10 May 2021.

### Data sources and integration

**Figure 1:**
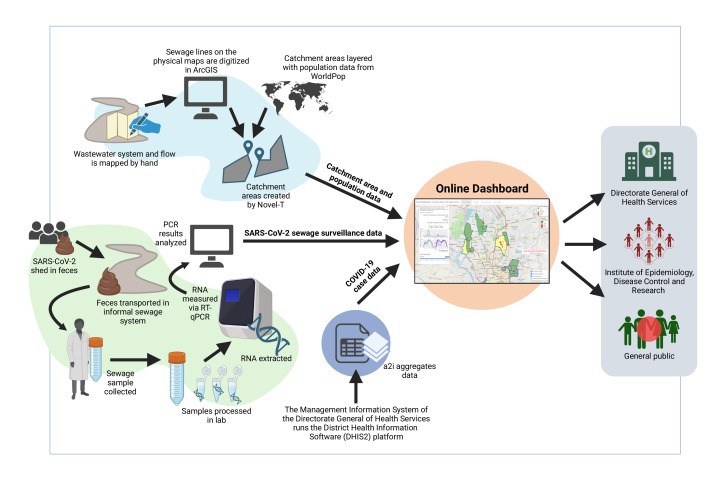
Data concept map for the SARS-CoV-2 Sewage Surveillance dashboard in Dhaka, Bangladesh. Estimated sewage catchment areas, population data, clinical COVID-19 case data and SARS-CoV-2 sewage surveillance data are integrated into the online dashboard and made available to public health officials, government stakeholders and the general public in real-time. This figure was created with BioRender.com. a2i, Aspire 2 Innovate; RT-qPCR, reverse transcriptase quantitative PCR.

Three main data sources are used in the dashboard visualisation: (1) sewage site coordinates, their estimated catchments and study area shapefiles, (2) weekly viral load of SARS-CoV-2 per sewage site and (3) reported incidence of COVID-19 cases (figure 1).

Sewage collection sites were opened progressively following detailed mapping of the informal sewage network and site selection.^8^ Unlike some high-income settings, maps of the sewage network and sewage catchments were not available prior to the study. Estimates of site catchment areas were provided by Novel-T. Population density data were obtained from WorldPop^16^ and used to estimate catchment populations as the most recent census occurred in 2011. Testing of sewage for SARS-CoV-2 began in March 2020 for sites in wards 8, 9 and 10 of the Dhaka North City Corporation (DNCC). The first positive detection occurred on 24 March 2020. Wards 2, 3 and 5 in DNCC commenced sampling in November 2020, and DNCC wards 18 and 19 commenced in December 2020. Finally, the study expanded to include 18 sites in the Dhaka South City Corporation from 15 May 2022. Sewage samples were collected weekly and tested for SARS-CoV-2 burden by icddr,b researchers by reverse transcriptase quantitative PCR. Estimates of viral load were then integrated into the dashboard within 5 days of sample collection.^8^

COVID-19 clinical incidence data from the 21 study wards included in the dashboard were provided on a weekly basis by the Aspire 2 Innovate (a2i) programme of the Information and Communication Technology Division of the Ministry of Posts, Telecommunications and Information Technology, Bangladesh. a2i receives case data from the Bangladeshi government via the District Health Information Software V.2 platform run by the Management Information System of the Directorate General of Health Services and geolocates the cases numbers by ward.

Detailed methods for sewage mapping, catchment area calculations, wastewater collection, sample testing and statistical analysis are outlined in our primary study paper published earlier.^8^

## Dashboard product

The dashboard was designed to give users a snapshot summary of the COVID-19 situation in Dhaka and to allow users to interactively explore the data at different geographical levels (ward and site). The dashboard tool https://dhakaesforsars-cov-2.research.virginia.edu/ was developed in RShiny,^17^ published on the shinyapps.io server^18^ and casted to a webpage developed by the UVA Custom Applications & Consulting Services (CACS). The code includes automated cleaning and processing steps which organise the data into a suitable format that can be presented on the dashboard maps, time series and tables. The dashboard is updated on a weekly basis and reported to the Bangladesh COVID-19 national task force to provide a comprehensive picture of COVID-19 in the Dhaka study area.

### COVID-19 mapper

**Figure 2:**
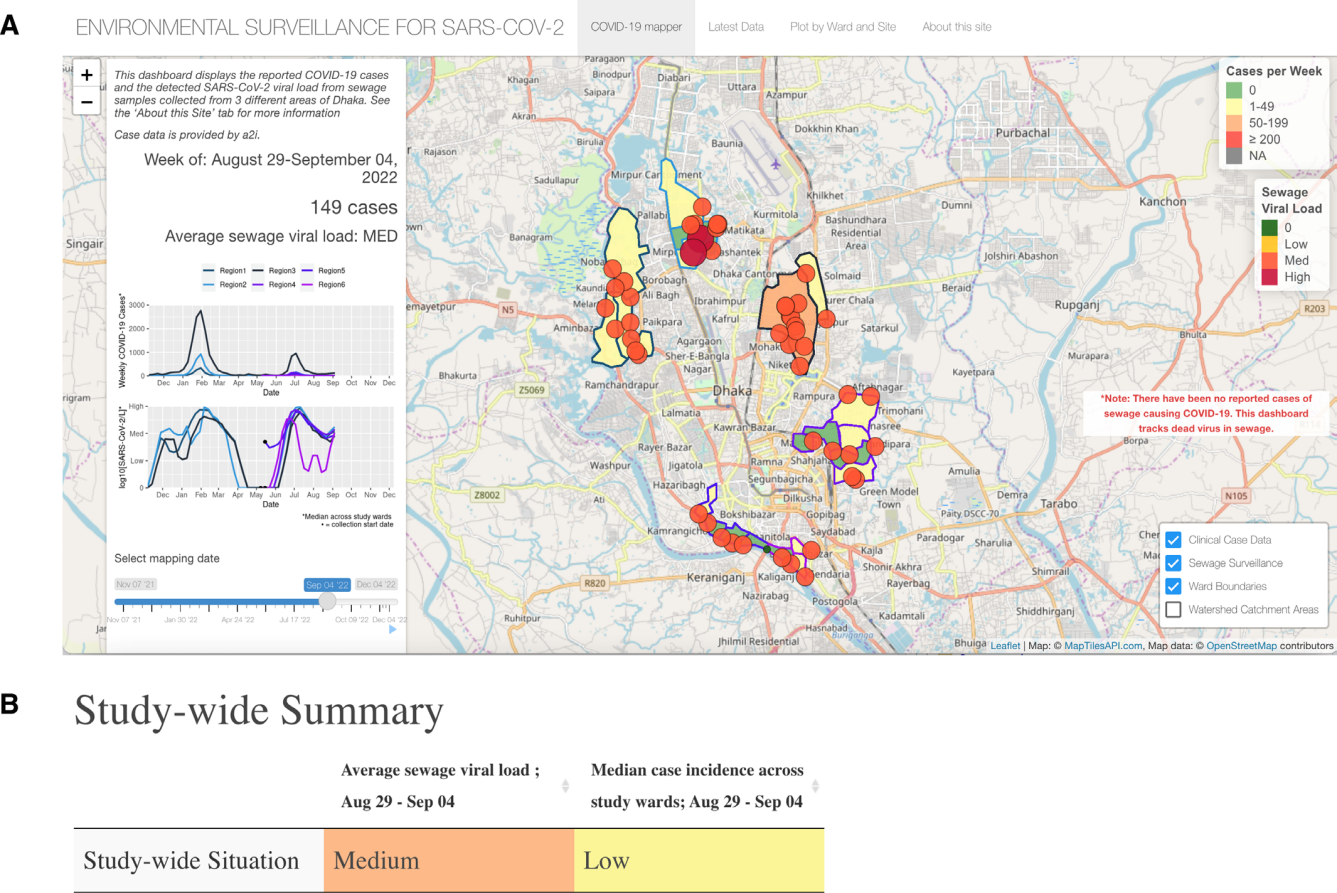
Study-wide features of the SARS-CoV-2 Sewage Surveillance dashboard in Dhaka, Bangladesh. (A) The homepage of the dashboard, which features the COVID-19 mapper tab that provides a geospatial study-wide summary. The white left side panel shows time series of both the weekly median SARS-CoV-2 sewage viral load and the summed weekly clinical case incidence per study region. The user can drag the timeline slider to view different weeks throughout the pandemic or press the play icon to watch the homepage step through each week. On the map, circles represent the location of each sewage collection site in the study area. The change in the radius and colour indicates the severity of the sewage viral load at that site. Outlined polygons represent each ward where clinical case incidence is reported. The shading of each ward indicates the severity of the case incidence in that ward. Wards are outlined in shades of blue and purple to categorise by study region. MED, Medium; (B) Study-wide data table showing categorisation of the average (mean) sewage viral load across all collection sites for a given week and the median case incidence across all wards for a given week. Data tables cells are colour coded in red, orange, yellow and green to represent high, medium, low and zero levels of the average sewage viral load and median case incidence. https://dhakaesforsars-cov-2.research.virginia.edu/.

The home page of the dashboard features the COVID-19 mapper (figure 2A). For a given week, the main map shows the case incidence across study wards and the SARS-CoV-2 viral load at each sewage collection site. The weekly sewage viral load is represented by circles at each collection site with a respective radius and colour shading representing a simplified severity category (Negative (green), low (yellow), medium (orange) and high (red)) for quick interpretation of the data. The three positive categories are defined by dividing the highest sewage viral load value from the beginning of surveillance up to the current date across all sites into evenly spaced terciles, defined dynamically overtime, as the pandemic progressed in real-time. The weekly clinical case data is represented by colour shading of each ward using a similar binning system. Legends are posted on the map to help guide the user’s visual understanding of the data. Additionally, the panel bar on the COVID-19 mapper tab shows the weekly date, total number of cases for that week, the average sewage viral load across all sites for that week and time series plots that summarise the case and viral load data by region. The user can drag the scroll bar at the bottom of the panel to change the week of data that they wish to view, and the map updates to show the data for that week. The ‘play’ button can be pressed to easily view the spatiotemporal changes over time. The user can also hover over each ward and site to display specific information about that location and can toggle on the sewage catchment areas to see which population each site represents.

### ‘Latest data’ tables

**Figure 3:**
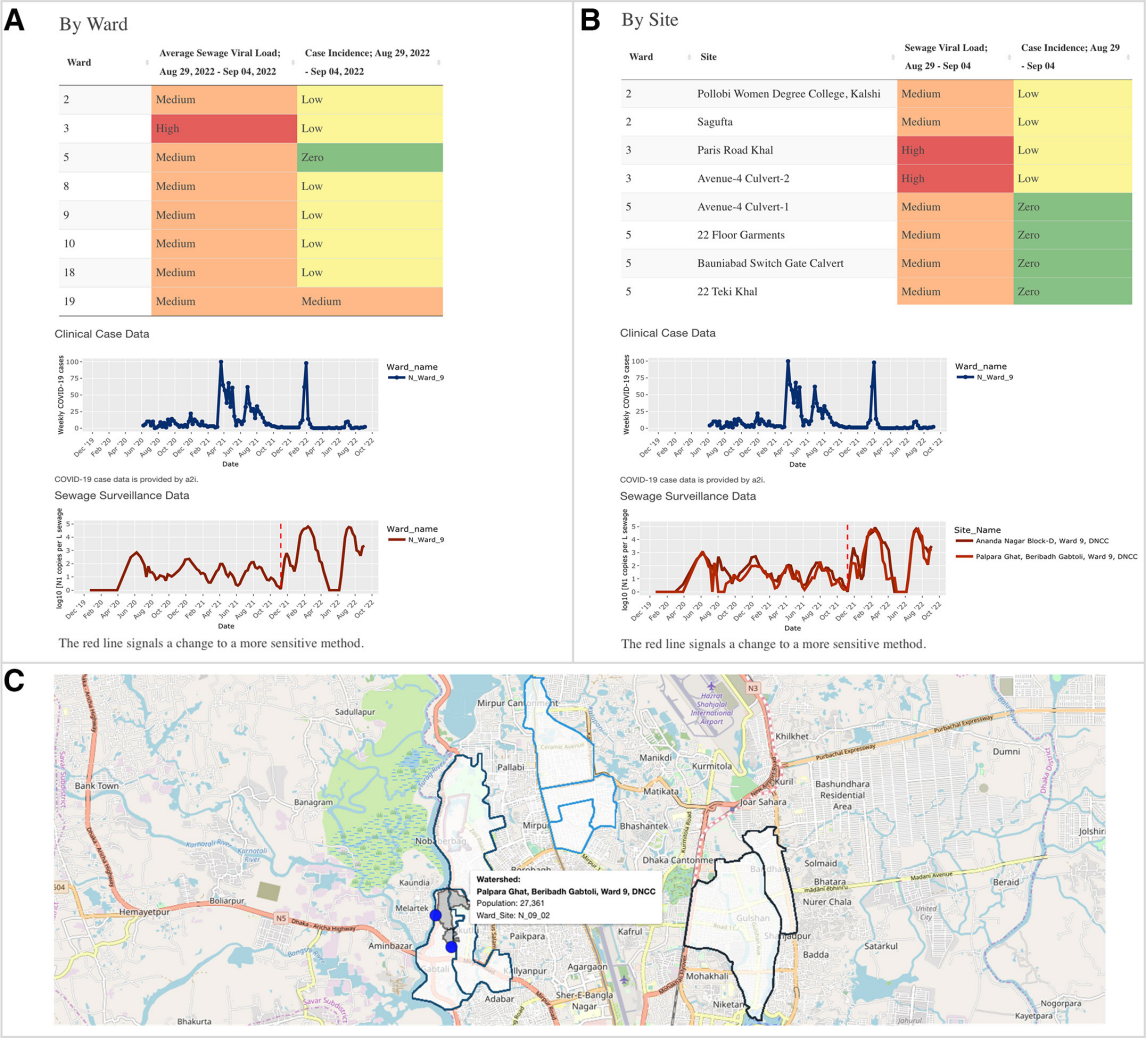
Features of the SARS-CoV-2 Sewage Surveillance dashboard in Dhaka, Bangladesh, on the ward and site level. (A) Dashboard data organised by ward. The data table shows the case incidence by ward for a given week and the mean sewage viral load across all collection sites in a ward for a given week, categorised by severity level (zero, low, medium, high). A time series is given for the respective ward for both the clinical data and the mean sewage viral load across the ward. (B) Dashboard data organised by sewage collection site. The data table shows the sewage viral load for a particular week detected at each site, categorised by severity level (zero, low, medium, high). The respective case data is reported at the ward level only. (C) The catchment area map displayed in the dashboard. Blue dots represent the collection site locations and the associated grey polygon represents the catchment area contributing to each collection point. Wards are labelled by N for Dhaka North City Corporation (DNCC) and S for Dhaka South City Corporation (DSCC). The longitudinal plots and catchment area map update as the user selects which ward and site data they want to visualise. https://dhakaesforsars-cov-2.research.virginia.edu/.

A simplified snapshot of the most recent clinical case data and sewage viral load is shown in the study-wide summary on the ‘latest data’ tab of the dashboard (figure 2B). This tab was made to have the most recent data easily accessible. The data tables create a side-by-side connection between the sewage trends and case trends, which has been helpful for public health officials to make quick inferences about the data. The interpretation tables on this tab categorise the sewage viral load and case incidence as either high, medium or low and colour codes each category according to the traffic light colour scheme. Partnered with the tables is a static map that displays the data from the most recent week. If the user wants to see how the data is stratified at the ward and site level, they can scroll down to view sewage viral load and case incidence by ward and by site (figure 3).

### 'Plot by ward and site' longitudinal plots

The ‘plot by ward and site’ tab is helpful for exploring the surveillance data over time at different spatial scales. The user can select from a drop-down menu which wards or sites they would like to view and can also choose to display the raw or smoothed data. Raw data refers to trend lines of the weekly data as it is reported while smoothed data refers to trend lines that are created by taking a moving average of the data over the three past weeks. Selections will automatically update the time series plots to show the temporal trends in clinical case incidence and sewage viral load at the select spatial scale. When the user selects specific wards or sites, the user can see a map with the estimated catchment areas plotted for their selection (figure 3C). Hovering over each catchment area will display information about the collection site name and estimated catchment population.

## Dissemination

The dashboard is updated on a weekly basis and reported to the Bangladesh COVID-19 national task force. Each week, the COVID-19 national task force met to compare different COVID-19 data sources including daily and weekly clinical case data, hospitalisations and vaccination coverage. This was complemented by the sewage surveillance dashboard for a better understanding of any gaps or limitations of clinical case data. Comparing clinical and sewage surveillance data facilitated the overall discussion for any need of localised implementation of non-pharmaceutical measures or increased clinical testing efforts. The dashboard has also been made available to the public through the IEDCR website: https://www.iedcr.gov.bd/ via their surveillance tab. Every year, the research team holds a dissemination meeting to explain the significance of our sewage surveillance to the public. The meeting is casted on public news stations and covered by local newspapers to create public awareness of the data collected from their communities.^19^

## A complementary tool for public health awareness

The final dashboard product has been an important tool for aiding public health and government officials throughout the COVID-19 pandemic. The dashboard has visualised data from 24 March 2020 to present day and has captured all of the SARS-CoV-2 variant waves. Displaying both clinical case data and sewage surveillance data in the dashboard allowed public health officials to know where under-reporting of clinical cases occurred.

**Figure 4:**
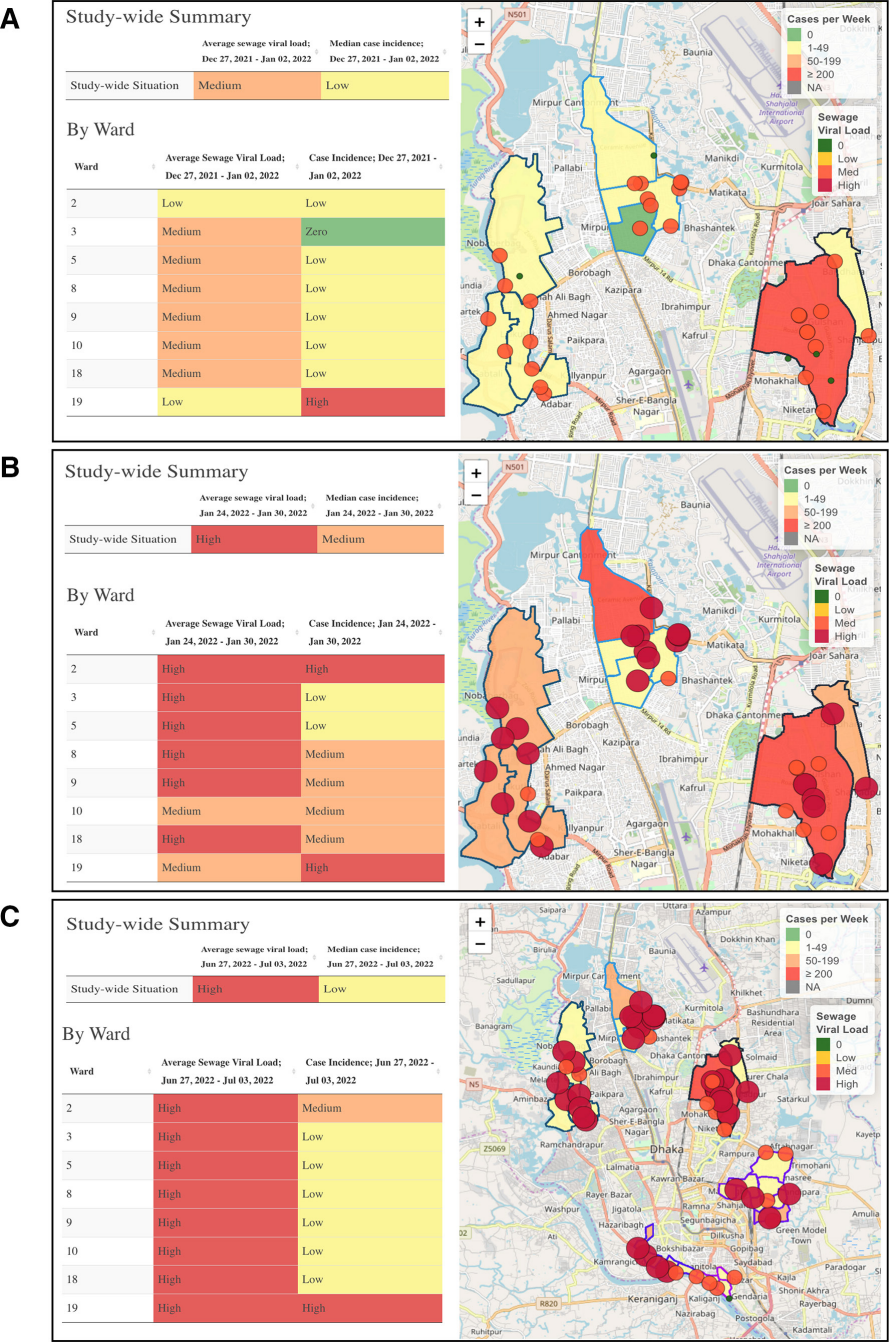
Visualisations of the SARS-CoV-2 Sewage Surveillance dashboard in Dhaka, Bangladesh, during three different periods of the COVID-19 pandemic. (A) Start of the Delta wave, week of 27 December 2021 to 2 January 2022, (B) peak of the Delta wave, week of 24–30 January 2022 and (C) peak of the Omicron wave, week of 27 June to 3 July 2022. Study-wide data tables show the categorisation of the average (mean) sewage viral load across all collection sites for a given week and the median case incidence across all wards for a given week, categorised by severity level (zero, low, medium, high). The ward data tables show the case incidence by ward for a given week and the mean sewage viral load across all collection sites in a ward for a given week, categorised by severity level (zero, low, medium, high). On the map, circles represent the location of each sewage collection site in the study area. The change in the radius and colour indicates the severity of the sewage viral load at that site. Outlined polygons represent each ward where clinical case incidence is reported. The shading of each ward indicates the severity of the case incidence in that ward. The visual comparison between SARS-CoV-2 sewage viral load and clinical case data shows how each data set reported differently throughout certain stages of the pandemic.

SARS-CoV-2 load in sewage increased up to 2 weeks before clinical cases during major waves of infection,^8^ and the dashboard clearly visualises this phenomenon. For example, at the beginning of the Delta wave in Dhaka, during the week of 27 December 2021 to 2 January 2022, the sewage viral load rose while the reported cases remained low (figure 4A). The sewage levels were ‘medium’ for most wards, while the case incidence was reported as zero or ‘low’ for all wards except ward 19, which has a high-income population and had access to a higher level of testing.^8^ Not until 3 weeks later, 24–30 January 2022, did the cases reach a substantially higher level and catch up with sewage levels (figure 4B), which supports the theory that sewage data can act as an early warning indicator for an impending rise in cases.

**Figure 5:**
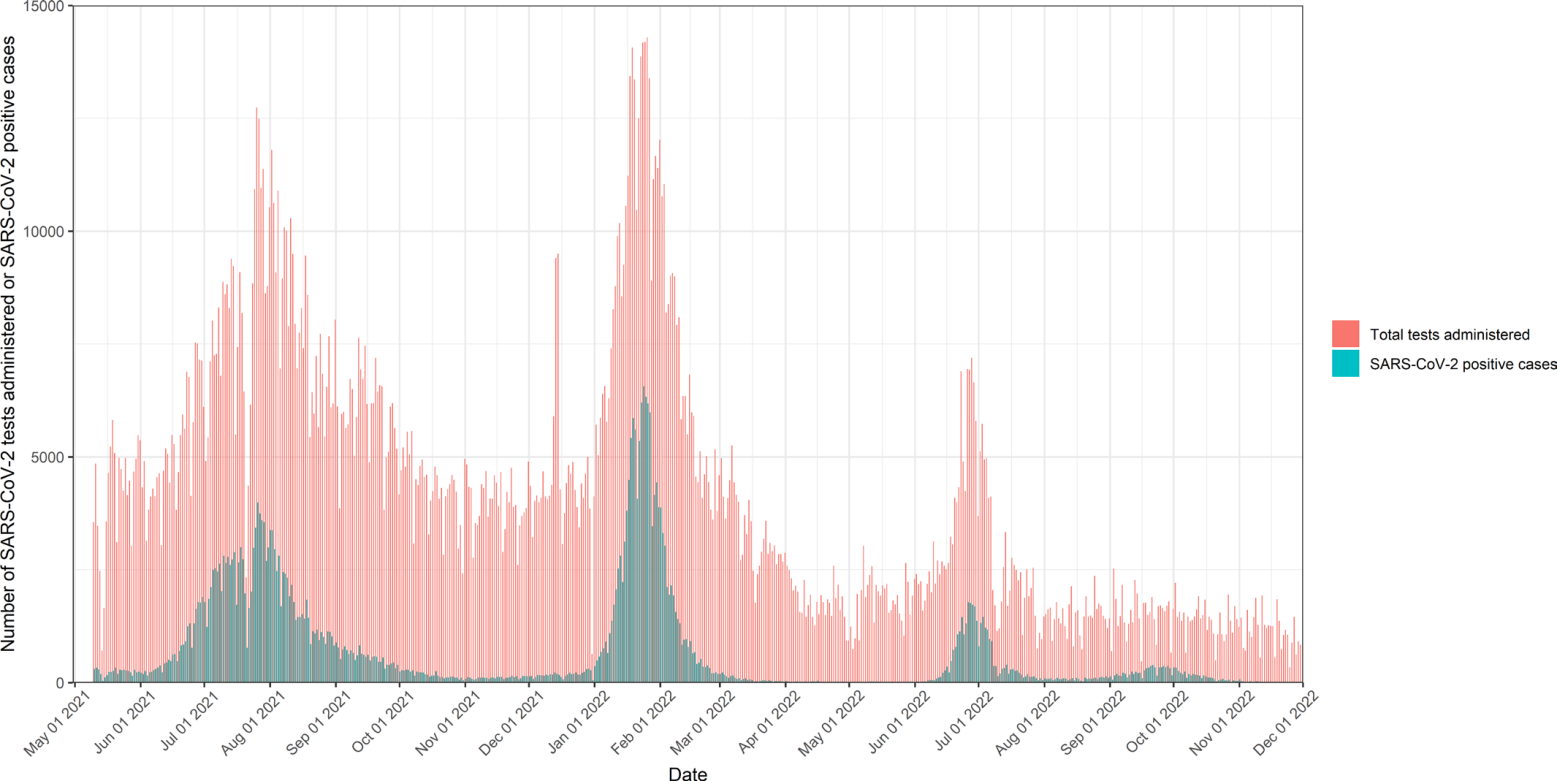
Daily number of SARS-CoV-2 cases reported (blue) and SARS-CoV-2 tests administered (red) in Dhaka city since 10 May 2021 to 20 November 2022 (last available date for total tests administered).

During the peak of the Omicron wave in Dhaka, from 27 June to 3 July 2022, viral load in sewage was categorised as high in every ward, while case incidence remained low for most wards (figure 4C). The dashboard visualisation of the Omicron wave highlights the value of pairing sewage viral load with clinical cases when case data is limited. The Omicron wave occurred during a time when fewer people were seeking testing (in part due to less severe symptoms and uptake in vaccination) (figure 5), so many infections were escaping the government reports. However, the high viral burden was still captured in sewage at a similar level to previous waves of infection.

Overall, the sewage surveillance dashboard was a supplementary tool for the discussion and decision-making of public health measures, especially when the clinical case data or testing was low. During the early period of the pandemic, the sewage surveillance dashboard helped identify hot spots. The fine spatial resolution of the sewage data by site and by ward guided authorities for increased testing in localised areas and helped guide the planning of vaccination campaigns.

## Successes and limitations

### Successes

The sewage surveillance dashboard for SARS-CoV-2 in Dhaka, Bangladesh, is one of the few publicly available dashboards that displays both SARS-CoV-2 sewage viral load data and COVID-19 clinical case data in a lower-middle-income country.^10 15^ We surveyed 70 dashboards outside of our study that report sewage surveillance around the world and found that 65 of the dashboards were reporting data for high-income countries.^15^ Of the five dashboards reporting from lower-middle-income and upper-middle-income countries, only one dashboard in Brazil showed both sewage and clinical case data. Our dashboard clearly depicts spatial variations in reported clinical incidence in contrast to more unanimous levels of SARS-CoV-2 in sewage.

Furthermore, the Bangladesh COVID-19 national task force has received weekly SARS-CoV-2 sewage data through the dashboard since 10 May 2021. From the general feedback from the national COVID-19 task force and IEDCR, we can conclude that public health officials have found it as an easy-to-use tool that aids in their assessment of the COVID-19 situation in Dhaka and has contributed to their decision-making. We have maintained lines of communication with the government end users of the tool and have received constructive input to improve the dashboard through its development.

### Limitations

One major barrier to the dashboard has been the scale and scope of clinical case data that we receive. Ideally, the dashboard would report the COVID-19 incidence data at the site catchment level so that the user could make granular comparisons with the sewage viral load data. In addition, incorporating the per capita clinical testing rate across different wards would provide information on the biases in clinical testing. However, this would only be possible if the location of all tests are geolocated, which has not been logistically possible. Nevertheless, sewage surveillance data provides site-specific information on SARS-CoV-2 transmission trends where clinical case data is limited.^6 7^ Several challenges to producing a dashboard in a lower-middle-income setting were overcome, such as the large effort to clearly visualise the population it represents by producing catchment areas of an informal sewage network. Hosting the dashboard server in Dhaka was also not a possibility due to capacity limitations and network setting issues. It was instead hosted on shinyapps.io and mirrored on a website created by the UVA CACS team, but we ultimately plan to host it on the IEDCR local server.

Further development of the dashboard should include COVID-19 variants of concern in sewage and clinical cases. This improvement can be made once testing for subvariants in sewage becomes more systematic in Dhaka. Furthermore, several enhancements could be made to the dashboard for public use such as including a translation to Bengali, the national language of Bangladesh, increasing the loading speed, making colour-blind adjustments and creating downloadable data and maps.

## Conclusion

In conclusion, presenting both sewage surveillance and clinical case data through a dashboard tool provides a comprehensive picture of COVID-19. This was especially true in Dhaka, a city in a lower-middle-income country, where clinical surveillance was limited due to lack of resources. Input from public health stakeholders during the development of the dashboard shaped it into a tool that was useful. Access to both sewage and clinical data at a fine spatial resolution helps to identify early warning signals in sewage and possible reporting biases in clinical data.

Although our dashboard was developed for the context of COVID-19 in Dhaka, many of the dashboard features are likely applicable for other settings where heterogeneities in clinical surveillance arise. Interpretation of sewage surveillance is particularly challenging in low-to-middle-income countries where informal sewage maps and site catchments are typically unavailable, and a strength of our dashboard was providing this information alongside the sewage surveillance data. We recommend that future dashboard developers present their clinical and sewage data on a fine spatial scale, design features that allow the user to make clear inferences and dynamically build their dashboards with consistent feedback from their end users. Similar dashboards could be created to concurrently track multiple infectious diseases to catch outbreaks early on and monitor pathogen burden in real-time.

## Data Availability

Data sharing not applicable as no datasets generated and/or analysed for this study.
